# Modulation of Redox Signaling in Chronic Diseases and Regenerative Medicine

**DOI:** 10.1155/2019/6091587

**Published:** 2019-04-23

**Authors:** Serena Zacchigna, Laura Sartiani, Claudia Penna, Gilda Varricchi, Carlo G. Tocchetti

**Affiliations:** ^1^International Centre for Genetic Engineering and Biotechnology (ICGEB), Trieste, Italy; ^2^Department of Medicine, Surgery and Health Sciences, University of Trieste, Trieste, Italy; ^3^University of Florence, Florence, Italy; ^4^University of Turin, Turin, Italy; ^5^Department of Translational Medical Sciences, Federico II University, Naples, Italy

Over the last decades, major advances in therapeutic strategies for chronic diseases have significantly reduced death rates. Redox signaling is being implicated in the pathophysiology of such diseases, affecting most organs. Proliferation of cancer cells, damage to cardiomyocytes and vascular cells, and exacerbation of inflammation are just a few examples of events importantly controlled by reacting oxygen species (ROS). Physiologic levels of ROS act as signaling molecules and modulate healthy functions, while high levels of ROS can derange the homeostasis of most organs and systems in our body. Hence, fine-tuning the complexity of redox signaling is a very up-to-date field of research.

Light-based technologies are emerging as powerful tools in several experimental and clinical arenas [1, 2]. Two manuscripts in this issue focus on the use of light to detect and interfere with ROS signaling. In their review, M. R. Antognazza et al. describe the state-of-the-art and recent advances in the field of photostimulation of oxidative stress (from photobiomodulation mediated by naturally expressed light-sensitive proteins to the latest optogenetic approaches) and highlight novel concepts based on optically driven ROS regulations mediated by polymeric materials.

The possibility to modulate oxidative stress in disease conditions also paves the way to innovative therapeutic intervention. The manuscript by K. Rupel et al. explores the changes in ROS levels upon photobiomodulation, also known as laser therapy, an emerging therapeutic option for patients affected by oral mucositis and other side effects of chemo- and radiotherapy. The authors have used multiple approaches to measure ROS levels, also including an elegant genetically encoded sensor, in both clinical samples and cultured cells. They showed that light variably impacts on intracellular ROS levels, depending on the used wavelength, prompting the use of a multiwavelength approach in the clinics.

The crosstalk between reactive oxygen species and NF-*κ*B (nuclear factor kappa-light-chain-enhancer of activated B cells) signaling is a consolidated knowledge in cancer development and progression. It is involved in crucial cellular events, such as apoptosis, differentiation, proliferation, angiogenesis, inflammation, and ROS production [3–13]. J. Gambardella et al. explore the effect of a small peptide able to mimic the minimum effective sequence of the RH domain of G protein-coupled receptor kinase 5 (GRK5), thereby inhibiting NF-*κ*B activity. This resulted in a potent suppression of tumor growth both in vitro and in vivo, suggesting a potential benefit in cancer treatment either alone or in combination with conventional anticancer therapies. In another interesting cancer study, M. Calvani et al. uncover a novel mechanism directed by mitochondrial ROS and *β*3-adrenoceptors to suppress the viability of cancer cells. In these cells, *β*3-adrenoceptors have a mitochondrial localization that enables them to drive mitochondrial dormancy and thus glycolytic metabolism, a documented proliferative advantage of tumor cells [14]. To lay the basis for this metabolic shift, *β*3-adrenoceptors upregulate uncoupling protein 2 (UCP2), reduce mitochondrial ATP and ROS synthesis, and increase cytoplasmic glycolytic enzymes. Notably, selective blockade of *β*3-adrenoceptor activity by SR59230A reverses the above effects causing a severe reduction of cancer cell viability, which is sustained by increased mitochondrial ROS. These results lead to the possibility of a selective antitumor therapeutic use of *β*3-adrenoceptor blockade.

Redox state and signaling crucially impact on almost all metabolic pathways. D-Tagatose is a sweetener, often found in dairy products, as it is 92% as sweet, but with only 38% of the calories of sucrose. Having shown a low glycation potential in phase 3 clinical trial, it appears particularly attractive for diabetic patients. Yet, no direct comparison between D-tagatose and other sugars has been performed so far. D. Collotta et al. compared for the first time the effect of D-tagatose and fructose on lipid and sugar metabolism. Using an in vivo approach, they found that fructose consumption led to increased body weight and abnormal glucose and lipid profile, associated with increased level of oxidative markers. Conversely, chronic overconsumption of D-tagatose in either liquid or solid formulation did not exert the same deleterious metabolic derangements. Thus, D-tagatose stands as a safer paradigm of sweeteners with limited toxicological impact on obesity and associated metabolic disorders, prompting the initiation of clinical studies to further confirm its safer metabolic profile in humans.

Another study by J. Gambardella et al. shows for the first time that parathyroid hormone (PTH) determines endothelial dysfunction in a ROS-dependent manner. This is particularly relevant as increased PTH activity is considered among the possible mechanisms increasing cardiovascular risk during vitamin D deficiency and in other pathological conditions. ROS-induced oxidation of both bradykinin receptor B2 and vascular endothelial growth factor (VEGF) receptor 2 emerged as a major effect of PTH in endothelial cells, likely resulting in impaired vasodilation and angiogenesis. The present work offers a novel and interesting model, supporting a relevant role of PTH in inducing endothelial dysfunction, which will need validation by in vivo experiments and in the clinics.

Aldosterone also has cardiovascular effects. A. Cannavo et al. reviewed the current knowledge concerning aldosterone actions in the cardiovascular system and the most recent preclinical studies and clinical trials designed to test better approaches aimed at countering the hyperactivity of the aldosterone/mineralocorticoid receptor signaling pathway in the setting of cardiovascular diseases.

In another metabolic study, M. M. Rahman et al. investigated the effect of loss of function of NADPH oxidase 2 (Nox2) on obesity-mediated alteration of bone remodeling in wild-type (WT) and Nox2-knockout (KO) mice fed with a standard lab chow diet (SD) as a control or a HFD as an obesity model. Indeed, there is evidence supporting the role of Nox in bone pathophysiology [15–18], and it is well known that a high-fat diet (HFD) generates obesity, with a negative impact on bone remodeling [19]. The authors demonstrate that HFD enhances bone mineral density to a greater extent in KO mice than in WT mice without affecting the total body weight and fat mass. HFD also significantly increases the number of adipocytes in the bone marrow microenvironment of WT mice as compared to KO mice. The bone levels of proinflammatory cytokines and proosteoclastogenic factors were also enhanced in WT-HFD compared to KO-HFD animals. Moreover, the in vitro differentiation of bone marrow cells into osteoclasts was significantly increased with bone marrow cells from WT-HFD mice as compared to KO-HFD mice, pointing towards Nox2 involvement in HFD-induced deleterious bone remodeling by means of increased bone marrow adipogenesis and osteoclastogenesis.

The paper of H. Jiang et al. investigates another metabolic condition, urolithiasis [20, 21], that is characterized by the development of stony concretions in the bladder or urinary tract. Solute carrier family 26 member 6 (Slc26a6), an important oxalate transport, is expressed mainly in the apical membrane of the intestine and kidneys. In this scenario, the authors demonstrate that downregulation of Slc26a6 expression attenuated ROS production to reduce crystal formation.

Interestingly, in the setting of chronic kidney disease, D. Cappetta et al. studied the inflammatory and oxidative responses regulated by DPP4 in a model associated to cardiovascular dysfunction (Dahl salt-sensitive rats). Inhibition of dipeptidyl peptidase 4 (DPP4) has emerged as a beneficial strategy against chronic diseases including type 2 diabetes and the related risk of kidney disease and other microvascular complications. In these settings, inflammation and oxidative stress play a major role and are controlled by DPP4 activity. In the kidney and many other organs, DPP4 degrades physiological substrates and is upregulated by pathogenic signals [22–24]. In this context, selective and chronic inhibition of DPP4 by sitagliptin reduced the progression of renal dysfunction, ameliorated markers of inflammation (reduced expression of NF-*κ*B, TNF*α*, IL-1*β*, IL-6, and MCP-1), induced macrophage polarization toward the anti-inflammatory M2 phenotype, and decreased NADPH oxidase 4 expression, oxidation of nucleic acids, lipids, and proteins. The protective effect of this approach supports the potential benefit of DPP4 inhibition in the context of chronic multiorgan diseases.

Huge research efforts are focusing on therapeutic strategies in respiratory diseases [25, 26]. A. G. Fois et al. conducted a study on idiopathic pulmonary fibrosis (IPF), a chronic lung disease characterized by enhanced fibrotic response [27] that can eventually also lead to pulmonary hypertension and right ventricular dysfunction [28, 29]. Molecular and cellular features characterizing the onset and progression of this devastating disease are not clear yet, but an aberrant remodeling of the pulmonary vasculature seems to be involved [30]. The authors showed that enhanced production of reactive oxygen species (ROS) induced by sera from IPF patients drives both collagen type I deposition and proliferation of primary human pulmonary artery smooth muscle cells (HPASMCs). These effects were significantly inhibited in cells treated with the NADPH oxidase inhibitor diphenyleneiodonium (DPI) proving the causative role of ROS and suggesting their potential cellular source. On the opposite, sera from IPF patients administered with pirfenidone did not increase ROS generation and collagen synthesis in HPASMCs, suggesting that antioxidant properties are involved in the in vivo effect of this molecule.

Finally, the review by E. Cianflone et al. summarizes our current knowledge on the mechanisms potentially affecting the regenerative potential of the aged heart [31, 32]. To what extent these events are specific for the heart or could be transposed to other organs, which similarly lose their regenerative capacity during the postnatal life, will be an interesting topic for future investigations.
